# Size matters: a review of the challenges in and importance of multimodal approaches to the management of patients with small, node-negative, triple-negative breast cancer

**DOI:** 10.3389/fonc.2025.1465147

**Published:** 2025-07-23

**Authors:** Vishnu Prasath, Briana To, Dionisia M. Quiroga, Bridget A. Oppong, Kai Johnson, Benjin Facer, Robert Wesolowski

**Affiliations:** ^1^ Department of Internal Medicine, The Ohio State University Wexner Medical Center, Columbus, OH, United States; ^2^ Division of Medical Oncology, The Ohio State University Comprehensive Cancer Center, Columbus, OH, United States; ^3^ Division of Surgical Oncology, The Ohio State University Comprehensive Cancer Center, Columbus, OH, United States; ^4^ Division of Radiation Oncology, The Ohio State University Comprehensive Cancer Center, Columbus, OH, United States

**Keywords:** triple-negative breast cancer, chemotherapy, radiation therapy, surgical oncology, multimodal management

## Abstract

Patients diagnosed with small (T1a-c) node-negative triple-negative breast cancer (TNBC) comprise an understudied population. These patients have been commonly excluded from participation in large, practice-changing clinical trials that establish improvements in disease-free and overall survival due to neoadjuvant or adjuvant systemic therapies as well as innovative local therapies. Despite this, patients with small, node-negative TNBC are at higher risk for early relapse compared to patients with hormone receptor-positive breast cancer matched for the same T and N stage. We highlight retrospective and prospective studies that analyze the benefit of chemotherapy in small node-negative TNBC patients. Furthermore, we discuss current guidelines for radiation therapy, surgical management, and relevant studies examining local therapy for patients with early-stage node-negative TNBC.

## 1 Introduction

Triple-negative breast cancer (TNBC) by clinical definition lacks estrogen receptor (ER) expression, progesterone receptor (PR) expression, and HER2-neu overexpression. Despite this uniform definition, TNBC represents a heterogeneous group of tumors that tend to recur earlier than other tumor subtypes and with a high propensity of impacting African American populations, those with germline BRCA1 mutations, and women less than 50 years of age (1–4). TNBC carries the worst prognosis when compared to other breast cancer molecular subtypes due to a high risk of early relapse and mortality (1, 5, 6). TNBC cases constitute about 15-20% of all breast cancers but account for about one-third of all breast cancer mortality (7). This subtype has the most limited effective targeted treatment options, which have only recently started to include novel small-molecule inhibitors, antibody-drug conjugates, and immunotherapy (1, 8).

Small (T1a-c), node-negative breast cancers make up less than 20% of all breast cancers across the globe (9). Based on American Joint Committee on Cancer (AJCC) staging version 8, T1mi tumors are those measuring 1mm or less; T1a are tumors larger than 1mm and smaller than 5mm; T1b are tumors measuring between 6-10mm; and T1c are tumors measuring between 11–20 mm. Small (T1mi-c), node-negative breast cancers constitute an understudied treatment population, as these tumors have been excluded from a variety of randomized clinical trials, especially those studies testing the addition of systemic therapies in neoadjuvant or adjuvant settings (10). Therefore, questions regarding optimal treatment modalities remain largely unanswered in this subgroup in comparison to the higher-risk groups (10). Clinical trials have shown that TNBC is sensitive to chemotherapy; therefore, it constitutes a cornerstone in the standard of care for this aggressive breast cancer subtype (6). Due to its aggressive nature, the threshold to offer neoadjuvant or adjuvant chemotherapy to node-negative patients is typically lower in TNBC as compared to patients with hormone-sensitive breast cancer. However, the minimal tumor size in which patients with node-negative TNBC should receive chemotherapy is controversial and lacks strong clinical evidence from randomized clinical trials.

Based on the National Comprehensive Cancer Network (NCCN) guidelines, no adjuvant systemic chemotherapy is recommended for pT1aN0 tumors. For pT1bN0 tumors, adjuvant therapy can be considered, while adjuvant therapy is recommended for pT1cN0 tumors (11). The 2023 European Society for Medical Oncology (ESMO) Clinical Practice Guidelines for Early Breast Cancer detail its recommendations for small node-negative TNBC and recommend considering no adjuvant systemic therapy for T1a tumors after surgery and 6–8 cycles of adjuvant systemic chemotherapy for T1b tumors after surgery (12). Finally, for T1c tumors, these guidelines recommend neoadjuvant chemotherapy (NACT) with a taxane and platinum backbone, followed by an anthracycline-containing regimen and surgery. In patients with evidence of residual breast cancer following NACT and surgery, there are additional recommendations for adjuvant systemic anti-cancer therapy (13–17). As treatment data for this narrow subset of aggressive and understudied breast cancers remains limited, we sought to explore the landscape of published literature and clinical trials testing local and systemic treatments in patients with small node-negative TNBCs.

## 2 Retrospective studies of systemic therapies

### 2.1 Benefit of adjuvant chemotherapy by tumor size

Numerous retrospective studies have examined the impact of adjuvant chemotherapy in node-negative T1 TNBC. Several of these studies have concluded that adjuvant chemotherapy in T1a and T1b TNBC did not confer any survival benefit. For example, a study by Ho et al. examined the impact of adjuvant chemotherapy on 5-year local recurrence-free survival and distant metastasis-free survival (MFS) in 194 patients with node-negative TNBC with tumors < 1 cm (18). In their cohort, 58% of patients received chemotherapy, which most commonly consisted of combined or sequential cyclophosphamide, methotrexate, and 4-fluorouracil. The median follow-up of this study was 73 months. There was no significant difference between 5-year locoregional recurrence-free survival (96.2% vs. 96%) and distant recurrence-free survival (95.9% vs. 94.6%) in patients with T1mic-bN0 TNBC who received chemotherapy compared to those who did not. When stratifying patients into T1mic/T1a and T1b subgroups, there was again no survival difference between patients who received chemotherapy and those who did not. Similarly, De Nonneville et al. examined the outcomes of 284 patients with node-negative T1a or T1b tumors across 15 French medical centers from 1987 to 2023 (19). They found no significant benefit associated with adjuvant chemotherapy as demonstrated by a hazard ratio (HR) of 0.77 (confidence interval (CI) 0.4-1.46, p=0.46) and a 5-year disease-free survival (DFS) of 90% in patients who received adjuvant chemotherapy vs. 84% in those who did not. There was no improvement in MFS with an HR of 1 (CI 0. 46-2.19, p=0.997) and a 5-year MFS of 90% in both groups. While both of these studies are limited by their sample sizes, a larger study derived from the Surveillance, Epidemiology, and End Results Program (SEER) database that was composed of 1739 patients with node-negative T1a TNBC diagnosed from 2010 to 2019 also demonstrated no significant benefit in overall survival (OS) (HR 0.63, CI 0.35-1.13, p=0.122) or breast cancer-specific survival (BCSS) (HR 0.95, CI 0.37-2.43, p=0.908) when chemotherapy was provided versus locoregional therapy alone (20).

In contrast to T1a and T1b TNBC, current NCCN guidelines support the use of adjuvant chemotherapy in node-negative T1c TNBC. Support for these guidelines can be demonstrated in several retrospective studies that have found a survival benefit associated with adjuvant chemotherapy in T1c tumors. An et al. conducted a retrospective study including 351 patients with T1 TNBC treated at Sun Yat-Sen University Cancer Center who most commonly received a combined anthracycline and taxane regimen (epirubicin, cyclophosphamide, and a taxane) as their adjuvant chemotherapy (10). In contrast to previous studies, results from this study demonstrated an improvement in relapse-free survival (RFS) for patients who received adjuvant chemotherapy (92.5%) as compared to those who did not (66.5%) (HR 0.19, CI 0.09-0.40, p<0.001). However, further subgroup analysis revealed a survival benefit only in the T1c group and not in the T1a or T1b subgroups. Another retrospective study conducted by Steenbruggen et al. evaluated adjuvant chemotherapy on OS and BCSS in 4366 patients with TNBC from the Netherlands Cancer Registry who were diagnosed between 2005 and 2015 (21). This study had an impressive median follow-up time of 8.2 years. In general, adjuvant chemotherapy was associated with improved OS (HR 0.58, CI 0.46-0.73, p<0.001) and BCSS (HR 0.65, CI 0.48-0.89, p<0.001) in T1N0 TNBC. However, when stratifying into various subgroups based on tumor size, the survival benefits with adjuvant chemotherapy were only significant in the T1c group. Adjuvant chemotherapy was not associated with better OS or BCSS in T1a and T1b tumors. Fasano et al. conducted a retrospective analysis of TNBC cases at Weill Cornell Medicine and Henry Ford Health System (22). The study included 258 patients with T1N0 TNBC who received adjuvant chemotherapy and included a median follow-up of 4.7 years. Similar to prior studies, adjuvant chemotherapy only improved the 5-year OS in patients with T1c tumors (93.2% vs. 75.2%, p=0.008). There was no significant difference in 5-year OS among patients with T1a-b disease who received chemotherapy. Finally, in a recent study, Carbajal-Ochoa et al. analyzed the benefit of adjuvant chemotherapy in patients with T1b and T1c TNBC utilizing the SEER database (22). This study examined OS and BCSS among 3388 patients with T1b and 8122 with T1c disease with a median follow-up of 66 months. After adjusting for demographic, clinicopathological, treatment, and socioeconomic covariables, chemotherapy was associated with improved OS in both the T1b (HR 0.52, CI 0.41–0.68, p<0.001) and T1c (HR 0.54, CI 0.47–0.62, p<0.001) groups. Interestingly, there was a significant difference in BCSS with chemotherapy only in patients with T1c (HR 0.79, CI 0.63–0.99, p=0.043) disease and not with T1b disease (HR 0.70, CI 0.45–1.07, p=0.10). Overall, this study demonstrated that adjuvant chemotherapy may be beneficial in those with T1c disease, while it did not confer an improved BCSS, but showed better OS, in T1b disease. **Table 1** summarizes retrospective studies that examined the benefit of adjuvant chemotherapy in patients with small, node-negative TNBC.

**Table 1:** Retrospective studies- adjuvant chemotherapy in small node negative TNBC.

Study	Year	Sample size	Data source	Median follow up	Chemotherapy	T1a	T1b	T1c
Ho et al. (18)	2012	**194** T1mi + T1a (n=65) T1b (n= 129)	Memorial Sloan Kettering Cancer center database	73 months	- Anthracycline and taxane (25%) - Anthracycline (12%) - CMF/MFL (57%) - Other (6%)	*5-year DFS w/chemo 100%, w/o chemo 91.6%	5-year DFS w/chemo 94.7%, w/o chemo 97.2%	N/A
De Nonneville et al. (19)	2017	**284** T1a (n=78) T1b (n=200)	15 French medical centers	48.23 months	Not reported	5-year DFS w/chemo 90% (CI 81-94%), w/o chemo 84% (CI 74-90%) 5-year MFS w/chemo 90% (CI 81-95%), w/o chemo 90% (CI 83-95%)		N/A
An et al. (10)	2020	**351** T1a (n=19) T1b (n=67) T1c (n=265)	Sun Yat-Sen University Cancer Center	68.5 months	- CMF (6%) - Anthracycline (38%) - Taxane (10%) - Anthracycline & taxane (45%) - Platinum (1%)	5-year RFS w/chemo 92.3%, w/o chemo 100%	5-year RFS w/chemo 91.4%, w/o chemo 90%	5-year RFS w/chemo 92.8%, w/o chemo 47.2%
Steenbruggen et al. (21)	2020	**4366** T1a (n=284) T1b (n=923) T1c (n=3159)	Netherlands Cancer Registry	8.2 years	- Anthracycline & taxane (44%) - Anthracycline (13%) - Taxane (8%) - Unknown regimen (35%)	OS HR 3.52, CI 1.02-12.14 BCSS HR 4.28, CI 1.12-16.44	OS HR 0.90, CI 0.48-1.66 BCSS HR 1.12, CI 0.51-2.49	OS HR 0.55, CI 0.43-0.69 BCSS HR 0.60, CI 0.43-0.82
Fasano et al. (22)	2022	**282** T1a (n=36) T1b (n=85) T1c (n=137)	Weill Cornell, Henry Ford Health System	4.7 years	Not reported	5- year OS w/chemo 100%, w/o chemo 100% (p=0.3778)	5- year OS w/chemo 100%, w/o chemo 95.8% (p=0.2362)	5- year OS w/chemo 93.2%, w/o chemo 75.2% (p=0.008)
Bravo-Solarte et al. (20)	2023	**1739** T1a (n=1739)	SEER	51 months	Not reported	OS HR 0.63, CI 0.35-1.13, p=0.122 BCSS HR 0.95, CI 0.37-2.43, p=0.908	N/A	N/A
Carbajal-Ochoa et al. (60)	2023	**11,510** T1b (n=3,388) T1c (n=8,122)	SEER	66 months	Not reported	N/A	OS HR 0.52, CI 0.41-0.68, p<0.001 BCSS HR 0.70, CI 0.45-1.07, p=0.10	OS HR 0.54, CI 0.47-0.62, p<0.001 BCSS HR 0.79, CI 0.63-0.99, p=0.043

*Includes T1mic + T1a tumor. Bold value indicate total sample size. N/A, Not applicable.

### 2.2 Neoadjuvant vs. adjuvant chemotherapy

Studies that have examined the survival benefit of chemotherapy in small node-negative TNBC have primarily focused on chemotherapy in the adjuvant setting. However, there are several advantages and disadvantages of administering chemotherapy before breast surgery (also known as neoadjuvant chemotherapy, or NACT). The biggest advantage is assessment of pathologic response to NACT at the time of the definitive breast surgery. Patients who achieve complete pathologic response have significantly better disease-specific and overall survival (23). The biggest disadvantage of neoadjuvant chemotherapy (especially in patients with small, clinically node-negative tumors) is the risk of overtreatment. To determine if there is a difference in OS associated with upfront surgery followed by adjuvant chemotherapy compared to NACT followed by surgery, Huang et al. designed a retrospective study using the National Cancer Database (24). This large-scale study consisted of 35,521 patients who were diagnosed with T1N0 TNBC between 2006 and 2016. Patients who underwent surgery followed by adjuvant chemotherapy had a better OS compared to those who received NACT followed by surgery (5-year OS 90.5% vs. 88.1% respectively, p<0.001). This may be due to the fact that the adjuvant chemotherapy cohort had an increased frequency of radiation therapy and a lower frequency of T1c tumors (53.5%, in comparison to 70.7% in the NACT group) which may have played a role in the improved outcomes seen in this group. It is worth noting that age and comorbidity index were lower in the NACT group despite improved outcomes in the adjuvant chemotherapy group. Interestingly, when patients were stratified based on staging, patients with T1c disease who achieved pathologic complete response (pCR) after neoadjuvant therapy had improved 5-year OS compared to the upfront surgery and adjuvant group (94.4% neoadjuvant vs. 91.9% surgery/adjuvant, p=0.025). This difference was not observed in patients with T1a and T1b tumors. This increase in OS observed in T1c disease favors the use of chemotherapy in the neoadjuvant setting. In summary, the study showed that patients with upfront surgery followed by adjuvant chemotherapy do not have inferior outcomes to those who receive NACT followed by surgery. However, patients with T1c tumors who achieved complete pathologic response to NACT had the best outcomes, suggesting that neoadjuvant chemotherapy provides important prognostic information that can guide adjuvant treatments. In fact, based on the results of the CREATE-X and OlympiA trials, adjuvant capecitabine or olaparib (in patients with germline BRCA 1 or 2 mutations), respectively, leads to improvements in OS and invasive DFS in patients with early TNBC and evidence of residual disease following NACT (25, 26).

## 3 Prospective studies of systemic therapies

### 3.1 Platinum-based regimens

Preliminary data presented in 2022 from a phase III study by Gupta et al. shows that the addition of carboplatin to an anthracycline and taxane backbone for NACT led to an improvement in pCR, event-free survival (EFS), and OS seen primarily in patients under 50 with TNBC ranging from T1 to T3 and N0 to N1 (27). This study was conducted at Tata Memorial Centre in India and included 720 patients, with DFS as the primary outcome of interest. In this subpopulation of younger patients, 5-year DFS was significantly greater at 74.5% in the experimental arm and 62.3% (p=0.0003) in the control arm. Similarly, 5-year OS was also significantly greater in the experimental arm (76.8%) than in the control arm (65.7%) (p=0.0003). Furthermore, adding carboplatin to the anthracycline and taxane NACT backbone was shown to have a positive impact on OS with an HR of 0.75 (95% CI: 0.58-0.98, p=0.038) after adjusting for age and tumor size. While this trial included patients with stage I-III disease, it is one of the few TNBC prospective trials that included patients with small node-negative disease.

Conversely, Sharma et al. conducted a randomized phase II trial to compare anthracycline-free and anthracycline-containing neoadjuvant carboplatin regimens in patients with TNBC (28). The NeoSTOP trial tested an 8-cycle, 4-drug regimen consisting of carboplatin and paclitaxel followed by doxorubicin and cyclophosphamide, and compared it to a 6-cycle, 2-drug regimen of carboplatin and docetaxel. The trial found similar pCR rates and showed that EFS and OS were comparable between trial arms at a median follow-up of 38 months (28). This 100-patient trial, which included patients with stage I-III disease (T1c or greater), found the anthracycline-free NACT regimen including carboplatin to be an effective alternative to the anthracycline-based regimen studied. Unsurprisingly, the 2-drug regimen was associated with a more favorable toxicity profile. Only 19% of the included patients had T1c disease, and 30% of the sample population had nodal metastases, limiting interpretation of the validity of this regimen in patients with small node-negative disease. The authors note in their discussion that the inclusion of the lower-risk tumors could have skewed pCR rates; therefore, the authors conducted secondary analyses that excluded patients with stage I disease to ensure that their results were concordant with other recently published neoadjuvant studies. Notably, multivariate regression analysis did not find T stage to be associated with EFS or OS outcomes. Univariate analyses found that the only variable predictive of pCR was grade III disease on histology, while TNM stage and node status were not found to be significant. Additional analysis of patients with stage I tumors was not conducted in this trial (likely due to a small sample size), which limits the potential impact on treatment recommendations for small node-negative TNBC tumors.

### 3.2 Immunotherapy

While paradigm-changing in the TNBC field, the Keynote-522 trial is noteworthy in that it did not include patients with stage I disease (29). This trial played a big role in the US Food and Drug Administration’s (FDA’s) approval of pembrolizumab (an IgG4 antibody targeting an inhibitory program death-1 [PD-1] receptor on T lymphocytes) in conjunction with neoadjuvant multi-agent chemotherapy for early-stage TNBC. Luckily other trials were conducted around the same time showing improvements in patient outcomes with the usage of immunotherapies in small node-negative disease.

Sharma et al. studied the addition of neoadjuvant pembrolizumab to a neoadjuvant carboplatin and docetaxel regimen in the NEOPact trial, which included 115 patients with stage I (T1c)-III TNBC tumors (30). The trial showed an overall pCR of 58% with estimated 3-year EFS rates of 98% in the pCR group and 68% in the no-pCR group. The majority of patients included had stage II-III disease, limiting the application of this study to the stage I TNBC population. In fact, only 14 patients (12%) were TNM stage I. However, these results seem to indicate the possibility that anthracycline de-escalation with the addition of pembrolizumab is safe, particularly in patients with lower-risk disease. Of note, the pCR rates of stages I, II, and III disease were 69%, 58%, and 43%, respectively. Furthermore, in univariate analysis, only T stage (HR 5.22; 95% CI 1.74-15.67, p=0.01) and nodal status (HR 6.33; 95% CI: 1.74-23.05, p=0.001) were associated with reduced EFS. Since stage I TNBC cases were not included in Keynote-522, as previously mentioned, these early results showing the potential of pembrolizumab in an anthracycline-free regimen for stage I tumors constitute an exciting and eagerly awaited finding for the small node-negative TNBC population. While further randomized studies enrolling a larger proportion of patients with small, node-negative TNBC are needed to confirm these findings, the results of the NEOPact trial provide emerging evidence that the use of chemotherapy de-escalation with the addition of an immune checkpoint inhibitor is feasible and can be employed in further investigations targeting this patient subpopulation.

GeparNuevo was a phase II study in which 174 patients with cT1b-cT4a-d disease were randomized to receive either durvalumab or placebo NACT. The study showed a modest and statistically insignificant increase in pCR by about 9% and a significant increase in invasive DFS, distant DFS, and OS with the addition of durvalumab (31, 32). Sixty-one (35%) patients enrolled in this study had stage I disease, and 78 (44.8%) patients had T1b-c disease. OS rates were 95.2% and 83.5% in the durvalumab and placebo cohorts, respectively. In the pCR group, DFS rates were 95.5% and 86.1% in the durvalumab and placebo cohorts, respectively. Secondary analyses showed that the benefit associated with durvalumab was independent of the pCR status of tumors in this study. The study additionally identified a change in tumor-infiltrating lymphocytes (TILs), which appears to have predicted pCR in these patients. Interestingly, stage I tumors had an odds ratio (OR) of 0.813 (CI:.297-2.23) with regard to pCR, while stage IIA and greater tumors had an OR of 1.97 (CI: 0.932-4.17). Given the small patient number, this finding did not reach statistical significance; however, these results suggest that the benefit from the addition of durvalumab to NACT could be independent of tumor size. One limitation of this report was the fact that the study investigators did not perform separate analyses in T1a, T1b, and T1c patients, most likely due to very small patient numbers. Phase III randomized clinical trials are needed to further explore the effect of durvalumab in the neoadjuvant treatment of TNBC.

## 4 Radiation therapy

Radiation therapy (RT) is a key component of TNBC treatment (33). Though RT de-escalation is an active investigational area in several breast cancer settings, TNBC has traditionally been excluded from these trials due to its aggressive nature (11, 34). Clinical data have consistently demonstrated that most patients with TNBC benefit from adjuvant RT, including those with early-stage disease.

Breast conservation therapy (BCT) typically includes breast-conserving surgery (BCS) followed by adjuvant RT in the form of whole-breast irradiation (WBI) or accelerated partial-breast irradiation (APBI). In contrast to T1N0 HR+ breast cancer, where 5 years of endocrine therapy can replace adjuvant RT for some elderly patients, current NCCN guidelines recommend adjuvant RT for all TNBC patients who undergo BCS (11). Two population-based studies demonstrated that patients aged 70 or older with T1N0 TNBC experience worse OS when adjuvant RT is omitted after BCS (34, 35). WBI is the standard RT option in this setting. A tumor bed boost is also recommended, as it helps reduce local recurrence (36, 37). The addition of regional nodal irradiation (RNI) should also be considered for patients with T1N0 TNBC who have central/medial tumors (11). As ER- status is a “cautionary” pathologic factor for APBI (38), APBI may be considered for a patient with T1N0 TNBC, though supporting data are sparse. Small patient numbers and short follow-up times indicate a need for caution and further prospective data when considering APBI for patients with TNBC off-trial (37). One benefit of APBI – shortened treatment time – may be achieved with the 5-fraction WBI technique from the FAST-FORWARD trial (39). This trial was a multicenter, phase III randomized trial that examined a 5-fraction schedule of adjuvant WBI for patients with early breast cancer. Results from this study, in which 9.6% of patients in the experimental arm had TNBC, suggest that a shortened treatment time with 26 Gy in 5 fractions over 1 week was non-inferior when compared to 40 Gy in 15 fractions over 3 weeks. Additional studies will strengthen the validity of this assumption for patients with TNBC.

As in many other breast cancer subtypes, BCT is not inferior to mastectomy for early TNBC. The Memorial Sloan Kettering retrospective experience of 1242 patients with T1-2N0 TNBC treated with either mastectomy or BCT demonstrated 5-year locoregional relapse (LRR) of 5.4% and 4.2%, respectively (40). Another retrospective study involving 468 patients with T1-2N0 TNBC found slightly worse LRR-free survival for patients who underwent mastectomy compared to BCT (90% vs. 96%, p=0.022) (41).

Treatment of pT1N0 HR+ breast cancer with mastectomy and surgical axillary staging typically obviates the need for adjuvant RT in the absence of high-risk features. However, current NCCN guidelines suggest that patients with pT1N0 TNBC should be considered for post-mastectomy RT when the tumor is central/medial, tumor size is 2 cm, or surgical margins are < 1 mm (11).

Early-stage TNBC patients are excluded from many contemporary clinical trials involving RT, but at least 2 ongoing trials focus on or allow patients with early TNBC. A phase II trial (NCT04807192) is examining whether preoperative vidutolimod (a toll-like receptor 9 agonist) injections and a stereotactic body RT (SBRT) boost can elevate TIL levels. Increased TIL density has been associated with increased rates of pCR and improved survival in patients with BC (42). The combination of vidutolimob and RT, both of which have immunostimulatory effects, may increase immunogenic activity, leading to a smaller primary tumor and decreased micro-metastatic burden, which is a concern in TNBC even at early stages (43, 44). SABR-CaRe is a phase II trial (NCT04959474) studying the effect of calorie restriction during neoadjuvant SBRT in breast cancer patients with tumors ≤ 3 cm, including those with TNBC. Giving a full course of RT before surgery may be advantageous due to a smaller, more defined treatment volume (45).”

In summary, current clinical data do not support the de-escalation of RT for patients with T1N0 TNBC. Improved biological classification of TNBC and a demonstration of immunostimulatory RT effects may identify subpopulations within TNBC that will benefit from alternative RT regimens, including escalation or de-escalation. Current standard-of-care recommendations support the use of adjuvant RT in all patients with T1N0 TNBC after BCS and some patients with high-risk features after mastectomy. These recommendations are associated with 5-year local control of at least 90-95% (40, 41).

## 5 Surgery

### 5.1 Surgical management of T1N0 TNBC

Studies including the landmark National Surgical Adjuvant Breast and Bowel Project (NSABP) B-04 and B-06 trials cemented BCS as an alternative to mastectomy for breast cancer management (46, 47). The decision to pursue BCS over mastectomy should depend on the ratio of tumor size in relation to overall breast size, multicentricity, and the ability of the patient to receive RT (48, 49). Histopathologic features such as tumor histology, grade, ER, PR, and HER2 status are not considered for surgical decision-making (48–51). However, given the poorer prognosis associated with TNBC patients, providers may harbor biases toward “more” surgery despite data showing no benefit. Supporting data demonstrated that patients with TNBC, for example, have an increased risk of local recurrence regardless of the surgical procedure performed (52). Two meta-analyses and several retrospective studies provide important data to address this concern in the context of contemporary breast cancer management accounting for tumor phenotype (52, 53). Lowery et al. conducted a systematic review of locoregional recurrence after breast cancer surgery looking at the impact of biomarker expression (52). Several studies have further demonstrated that more extensive local surgery in the form of mastectomy for TNBC does not improve locoregional control when compared to BCT, and some even suggest that lumpectomy with breast radiation achieves better results (53).

Just as the local treatment of primary breast tumors does not differ based on subtype, neither should the axillary surgery. Sentinel lymph node biopsy (SLNB) is recommended with axillary lymph node dissection (ALND) indicated based on those results (11, 54). The practice-changing shift from the American College of Surgeons Oncology Group Z0011 trial also includes TNBC (55). This study found that patients with cT1–2 breast cancer and 1–2 positive sentinel nodes following BCS with planned whole-breast RT had no OS or DFS benefit when undergoing ALND compared to the observation cohort. In addition, it was noted that 27% of patients who underwent ALND had cancer in non-sentinel lymph nodes (56). This suggests that some of the patients who underwent SLNB alone may have had residual non-sentinel node disease that was not resected, which did not result in a significantly worse survival. ER- and PR- tumors were included, comprising 15% of each treatment arm. Still, the authors state in their limitations that “not all biological subtypes are represented in large numbers.” Subsequent studies show that the adoption of the Z0011 criteria and then the criteria for After Mapping of the Axilla: Radiotherapy or Surgery (AMAROS) study supported use of SLNB alone in appropriate candidates regardless of molecular subtype (57, 58).

While the type of surgery is not controversial, the sequence of surgery and chemotherapy is the more disputed issue. With larger T1 tumors, specifically T1c tumor there can be consideration for NACT. These patients are typically operable, however there may be an advantage to delaying surgery (30, 31). As discussed in previous sections, the use of NACT allows for the ability to identify clinical evidence of disease response to chemotherapeutic agents. In situations where there is residual tumor following NACT, we have the opportunity to change the drug regimen and add additional systemic therapy in the adjuvant setting.

## 6 Predictive biomarkers & ongoing studies

ctDNA and circulating tumor cells have the potential to become powerful tools in the oncologist’s armamentarium by allowing for minimally invasive assessments to determine both clinical response and treatment efficacy. Liquid biopsies will allow for more accurate determination of biomarkers capable of guiding clinical decision making and the small, node negative TNBC is an area with great need due to the lack of robust literature for this patient population. An early study from Hall et al. showed identification of CTCs after NACT was associated with a reduced OS and RFS in patients with Stage I-III TNBC (59). Studies specifically for small, node negative TNBC are needed to confirm and expand upon these earlier findings.

TILs similarly hold great promise as a prognostic and predictive biomarker. Literature shows that patients with early stage (Stage I-III) TNBC who did not undergo adjuvant chemotherapy or NACT who had a greater number of TILs were associated with improved survival. In stage I patients in particular, patients with a TIL level of 50% or more had a 5-year RFS of 89% (95% CI, 86-93% and OS of 95%(95% CI, 92-97%) while patients with a TIL level of 30% and lower had a 5-year RFS of 73% (95% CI, 70-76% and OS of 82%(95% CI, 79-84%) (60).

There are several ongoing studies (**Table 2**) examining the use of novel agents and locoregional therapies for T1N0 TNBC management, as well as translational studies gathering data on TILs and circulating-tumor DNA (ctDNA), to help us better stratify risk for this unique patient population. Additionally, there is a DNA plasmid-based vaccine being studied in patients with stage IB-III TNBC disease (61). As is evident from the breadth of ongoing studies, a wide array of investigations in this previously understudied population seek to identify optimal neoadjuvant and adjuvant therapy options; their results are eagerly awaited.

**Table 2:** Table of active prospective clinical trials.

NCT	Trial phase (n)	Agents	Inclusion critria	Primary end point
NCT05949021	II (20)	Liposomal Doxorubicin and Carboplatin	Tumor size < 2.5cm and N0/N1mi TNBC	DFS
NCT04768426	II (25)	Adjuvant Capectabine	Stage I-III TNBC with residual disease	ctDNA levels
NCT03812393	II (27)	Neratinib	cT1c-2 and N0–1 TNBC	PCR and response rate
NCT05749575	II (28)	Chidamide and Paclitaxel	Any TNBC	PCR
NCT04427293	I (12)	Lenvatinib and Pembrolizumab	T1b-T3/N0-N3/M0	TILs presence
NCT03546686	II (80)	Cryoablation and Pembrolizumab	TNBC >= 1.0cm	EFS
NCT04296175	III (808)	Epirubcin + Paclitaxel vs ddEpirubicin + Paclitaxel vs ddEpirubicin + Paclitaxel + Carboplatin	TNBC node positive or node negative with Ki67 >=50%	DFS
NCT06230185	I (422)	N/A	Stage I-III TNBC	Correlation between Molecular Residual Disease and PCR
NCT05812807	III (1295)	Pembrolizumab	T1cN1–2 or T2-4N0–2 TNBC	RFS

N/A, Not applicable.

## 7 Discussion

There is a lack of robust, high-level clinical evidence surrounding the management of small node-negative TNBC, and very limited conclusions can be made based on the results of the few prospective studies that enrolled patients with stage I TNBC. However, there has been some recent progress in the field with the publication of several landmark studies.

Several retrospective studies have been conducted to better understand the role of chemotherapy in small node-negative TNBCs. Many suggest that adjuvant chemotherapy in patients with T1c TNBC appeared to improve long-term outcomes. In contrast, some studies have shown that T1a and T1b TNBC does not benefit from adjuvant chemotherapy. Interestingly, a study conducted by Carbajal-Ochoa et al. showed improved OS, but not BCSS, in T1b TNBC (62). The authors hypothesized the improved OS observed in the T1b group may be due to selection bias, given the fact that patients with comorbidities were less likely to receive adjuvant chemotherapy. Unfortunately, comorbidities were not accounted for in their analysis due to data availability. This study taken in context suggests little benefit with T1b TNBC despite improved OS seen in the article by Carbajal-Ochoa et al. because of some limitations such as selection bias (healthier patients with T1b tumor tend to be more likely to receive chemotherapy compared to patients with poorer performance status and co-morbid conditions). Therefore, definitive conclusions cannot be drawn from these results.

Although these retrospective studies may provide valuable clinical evidence, there are several limitations that need to be considered when interpreting their results. First, Fasano et al. included only patients from two academic centers (22); thus, it is unclear if the findings from these studies will be applicable to the general population. In addition, several studies have smaller sample sizes, making it more challenging to identify true differences between those who received adjuvant chemotherapy and those who did not. Primary endpoints were also inconsistent among different studies, so it is difficult to compare results across these studies – an important consideration when studies have conflicting results. Importantly, not all of the studies utilized multivariate analysis or propensity score matching to assess survival analysis, so the results of those studies may be affected by confounding variables. Furthermore, some of the studies lacked information regarding chemotherapy regimen, dosing, and chemotherapy discontinuation information, which may have affected the analysis of chemotherapy on survival outcomes. Given the variations of chemotherapy regimens utilized, it would be interesting to determine whether certain adjuvant chemotherapies can confer survival benefits even in T1a and T1b TNBC patients. In the same vein, it would be interesting to see how further classification of TNBC based on gene expression arrays in the future could guide treatment decisions in patients with small, node-negative TNBC. Unlike randomized controlled studies where patients are randomized to a treatment group, patients in retrospective studies received treatments that were carefully chosen for them based on other clinicopathologic factors that may not be included in the analysis. For example, patients who are younger and/or less debilitated are more likely to receive adjuvant chemotherapy (63, 64). This in turn can lead to selection bias since patients who did not receive adjuvant chemotherapy may have a higher risk of mortality due to other comorbidities and frailty. As such, future studies should be sure to document key covariates like comorbidities, frailty and chemotherapy dosing information so results can be compared across trials in the appropriate context.

In general, these retrospective studies support current NCCN recommendations to administer adjuvant chemotherapy to patients with node-negative T1c TNBC. Some studies do not demonstrate a significant survival benefit with adjuvant chemotherapy in node-negative T1a TNBC, while some studies showed some benefit from adjuvant chemotherapy (improved OS, but not BCSS), in the T1b group.

Some of the reported benefits of NACT include the ability to assess pathological response and determine pCR rates. pCR has become a vital element in the management of breast cancer as it has been shown to be directly associated with OS and DFS. Similarly, residual cancer burden (RCB) is another tool that offers greater granularity than pCR. RCB is calculated using a variety of pathologic factors to assign tumors to 1 of 4 classes ranging from RCB-0 (which is equivalent to pCR) to RCB-3 (extensive residual disease burden) (65). RCB has recently been shown to be prognostic across all breast cancer subtypes, with greater scores associated with reduced EFS. These metrics provide incredibly valuable clinical information only available after NACT and strengthen the utility of NACT in this population of patients.

The CREATE-X randomized trial established 6–8 cycles of capecitabine as beneficial in reducing recurrence risk and increasing OS in the population of patients who did not achieve pCR (25). The OlympiA trial showed that the addition of olaparib, a poly (ADP-ribose) polymerase (PARP) inhibitor, in those with HER-2-negative high-risk tumors with germline BRCA1 or BRCA2 pathogenic variants or likely pathogenic variants after neoadjuvant or even adjuvant therapy was associated increased DFS and OS (26). Therefore, even after the completion of neoadjuvant therapy, pCR and RCB metrics help in determining adjuvant therapy regimens; treatment regimens can be specifically tailored based on a wide variety of clinically relevant findings to improve survival. However, this has to be weighed carefully against the risk of overtreatment and the resulting short- and long-term toxicities of neoadjuvant chemotherapy, particularly in patients with small, clinically node-negative TNBC.

As far as local therapy is concerned, generally, the same principals guiding the choice of breast surgery, lymph node assessment, and radiation therapy in patients with more advanced-stage TNBC apply to patients with small, node-negative TNBC. Despite limited studies, current clinical data support the use of adjuvant RT for patients with T1N0 TNBC who underwent BCS as well as those with high-risk features post-mastectomy. A large retrospective study showed better OS and BCSS rates associated with adjuvant radiation therapy in patients aged ≥70 years with T1N0 TNBC (34). Similarly, another study demonstrated greater 5-year OS associated with adjuvant RT in patients ≥70 years old with T1-2N0 TNBC. This study, however, excluded patients <70 years old and did not stratify patients into subgroups of T1N0 TNBC (35). Both of these retrospective studies utilized large publicly available databases, thus certain confounding factors may not have been accounted for due to data availability. These studies highlight the need for prospective studies to examine the survival benefit of adjuvant radiation in early TNBC disease, especially in younger patients.

The literature has shown similar outcomes between BCS and mastectomy and notes the decision on surgery type should not depend on histopathologic features or tumor size (with some notable exceptions discussed previously). It appears that TNBC is associated with an increased risk of local recurrence despite surgical modality. In fact, mastectomy is not associated with improved locoregional control in comparison with BCS. However, the literature shows NACT can be considered in T1c tumors with reports of improved outcomes after NACT. Patients who are initially not candidates for BCS could become candidates if they experience tumor downstaging following neoadjuvant BC therapies. BCS has been shown to have improved quality-of-life outcomes over mastectomy, most notably with regard to body image and future perspective (66).

In summary, the available clinical evidence suggests that chemotherapy is beneficial in node-negative TNBC patients with tumors > 1 cm while controversy exists for patients with T1bN0 TNBC. Locoregional management of patients with small, node-negative breast cancer generally follows the same principals as those for patients with larger tumors. A variety of studies testing novel local and systemic approaches for patients in this subgroup are underway. Future research should incorporate risk stratification of patients with small, node-negative TNBC based on histologic, clinical, and molecular data.
